# Non-collinear magnetic configuration mediated exchange coupling at the interface of antiferromagnet and rare-earth nanolayers

**DOI:** 10.1038/s41598-022-26407-4

**Published:** 2022-12-17

**Authors:** Junyu Huang, Chang Liu, Yifan Cui, Yuxiang Ling, Keming Chen, Kunlong Zhao, Xiangshang Xiao, Bin Yuan, Amitesh Paul

**Affiliations:** 1 Department of Materials Science and Engineering, and Guangdong Provincial Key Laboratory of Materials and Technologies for Energy Conversion, Guangdong Technion-Israel Institute of Technology, 241 Daxue Lu, Shantou, 515063 Guangdong China; 2 Department of Materials Science and Engineering, Technion-Israel Institute of Technology, 32000 Haifa, Israel

**Keywords:** Materials science, Physics

## Abstract

Mn$_{3}$Ir/CoFe bilayer is a prototypical exchange-coupled antiferromagnet (AF)–ferromagnet (FM) system. Nevertheless, a strong exchange coupling between FM and rare-earth(RE) interfaces of Fe/Dy and Fe/Tb has been established earlier. Strong coupling at the FM–RE interface originates from the number of irreversible spins owing to the imbalance in the non-collinear configuration in RE. However, exchange coupling between AF–RE could not be established due to the minimal number of irreversible spins in AF and RE. A frustrated inter-domain magnetic interaction leads to the coexistence of spin-freezing-like ordering around the temperature range of helical spin modulation at the exchange-coupled interfaces of RE-based specimens. To overcome the lack of coupling between the AF–RE interface, we use a sandwich structure of AF–FM–RE layers (Mn$_{3}$Ir/CoFe/Dy) as we demonstrate establishing considerable exchange bias in the system. Changing the bias direction during field cooling introduces possible differences in non-collinear directions (helicities), which affects the number of irreversible spins and consequent exchange coupling differently for opposite directions. The non-collinear structures in RE are topologically stable; thus, their directions of orientation can be regarded as an additional degree of freedom, which can be manipulated in all-spin-based technology.

## Introduction

The exchange bias phenomenon is described as a form of a unidirectional magnetic anisotropy that arises due to the interfacial exchange coupling between a ferromagnet (FM) and an antiferromagnet (AF), which can effectively delay the superparamagnetic limit^1–6^. Apart from the prototypical AF–FM system, measurements of exchange bias induced in bilayers and multilayers with ferromagnets (FMs) and rare-earth (RE) can be used to investigate such magnetic states of the systems with different (low-high) magnetic anisotropies in the context of topological domain configuration. Internal interactions such as exchange, Ruderman–Kittel–Kasuya–Yosida (RKKY) or long-range dipolar interactions can be used to influence information processing via the spin degree of freedom. Additional involvement of magnetic anisotropy on top of these interactions can stabilize topological spin configurations like spin helices or vortices. The helical structures in rare-earth being topologically stable can be manipulated in all-spin-based technology.

Topology is a classification of shapes that can continuously be deformed into each other. In most cases, they are induced by chiral interactions between atomic spins in non-centrosymmetric magnetic compounds such as in skyrmions or in thin films with broken inversion symmetry. Homeomorphism can be described as a continuous function between topological spaces that has a continuous inverse function. Magnetic helices or non-collinear structures forming 2$\pi$-DWs within a multilayer are typical examples of such topologies where its shape protects it from trivial unwinding (non-trivial winding) and thus can be manipulated without an electric or magnetic field. The protection in helices are not induced by chirality but are stabilized by magnetic anisotropies, long-range interactions, and exchange interactions^7^.

Earlier, evidence of superimposed helical magnetic configurations or 2$\pi$ domain walls *within both* materials of FM–RE or RE–RE were found^6–9^. Interestingly, magnetic investigations had also revealed an exchange bias coupling with superparamagnetic (SPM) or super spin-glass (SSG) like behavior, attributed to spin-frustrated interfaces^9^. However, exchange bias coupling between the non-collinear or collinear spin texture of Mn$_{3}$Ir^10–13^ and the helical spin structure of RE was rarely investigated. This is due to the fact that the exchange coupling between AF–RE is very weak for the two highly anisotropic materials. The lowest-order uniaxial AF and RE sublattice anisotropy constants are $K_{{\textrm{RE}}}$ ($K_{{\textrm{Dy}}}\sim 1.7\times 10^{5}~{\mathrm{J/m}}^{3}$^14^) and $K_{{\textrm{AF}}}$ ($K_{{\mathrm {Mn_{3}Ir}}}\sim 6.2\times 10^{5}~{\mathrm{J/m}}^{3}$^15^), respectively. Nevertheless, a strong coupling was established extensively between the AF–FM^6^ and also between the FM–RE interfaces^7^. In REs like Tb or Dy, the presence of large orbital momentum leads to a strong spin-orbit coupling and larger magnetic anisotropy. The large difference in the spin-orbit coupling in AF and RE elements can have significant influences on the demagnetization processes as well. Aided by the RKKY interaction, the magnetic modulations usually propagate coherently over a long-range, even within the intervening nonmagnetic or magnetic layers.

**Figure 1:**
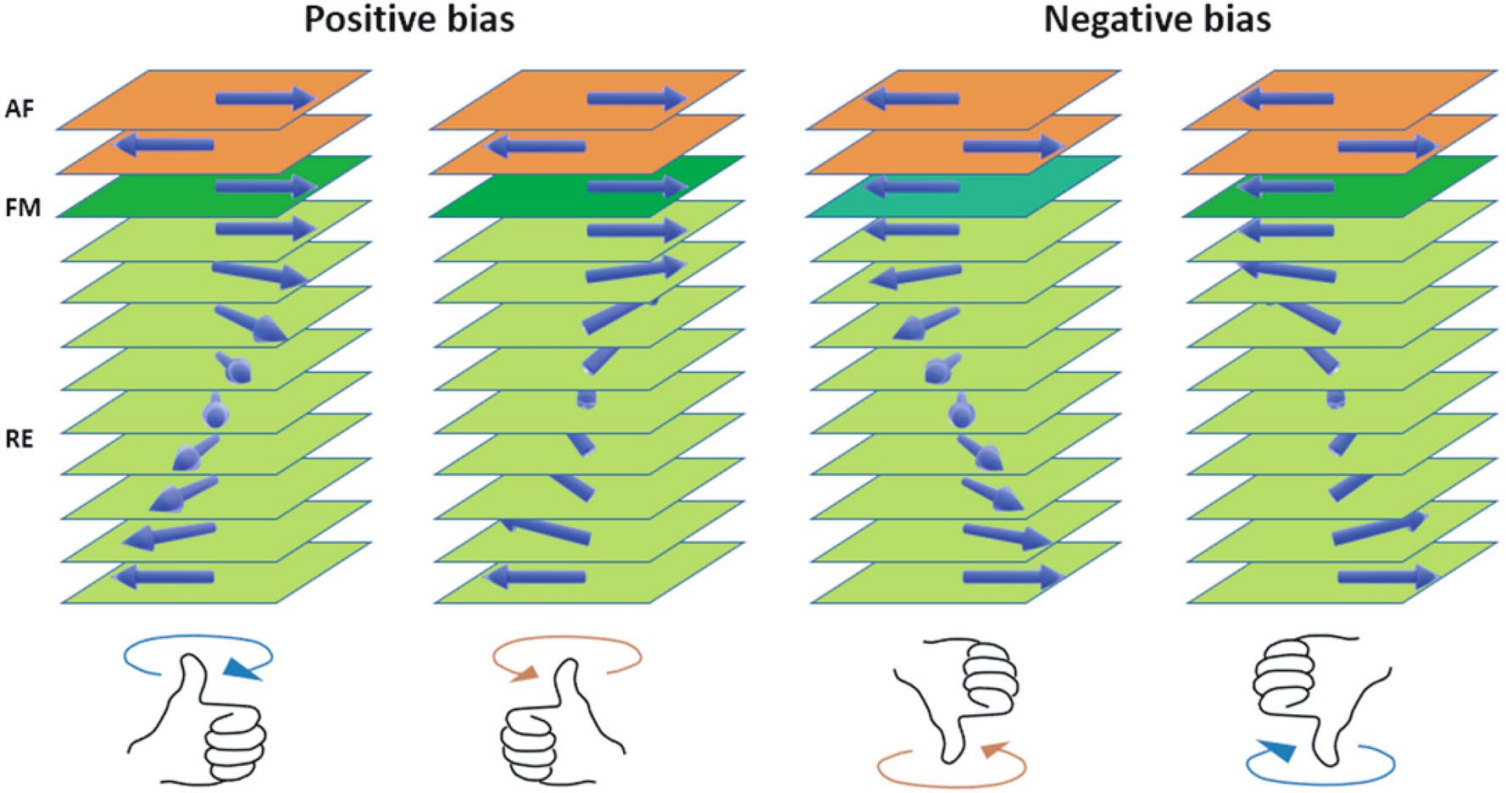
Sample sketch for *S*3. Considering a helical phase in Dy in *S*3, we show the sketch of the non-collinear spin configuration with different orientations. Different orientations can be induced upon a change in the field cooled direction from positive to negative bias. Such an inversion may lead to possible scenarios of helicity and concomitant chirality inversions. Magnetization measurements, however, cannot distinguish between helicities.

The helical structures in rare-earth are essentially due to spatially modulated magnetic states in systems with competing exchange interactions. Thus, an FM layer sandwiched between AF and RE materials would be interesting to explore as we expect an indirect coupling between AF and RE layers via an FM layer. Inversion of the helicity with associated handedness or chirality can thereby be tailored for the RE coupled to the FM simply by flipping the field cooling direction for the underlying AF–FM system. Inverted helicity represents a $\pi$ projection of the sublattice spin direction of motion, which undergoes a concomitant chirality ($\circlearrowleft \leftrightarrow \circlearrowright$) reversal. A sketch in Fig. 1 shows the possible scenarios of helical-chirality inversion. The scenarios in the figure, however, are possible cases when we consider the helical phase of the RE. Since incoherent domain structures are expected, we need not consider the helical phase for the AF-based systems in interpreting their magnetic behavior. The manipulation and/or control of domain dynamics within AF–RE systems can facilitate the design and construction of functional chiral nanomaterials as an additional degree of freedom in all-spin-based spintronics. The inter-sublattice exchange between RE and transition-metal spins is antiferromagnetic^16^.

Here, we investigate the magnetic properties of AF–FM, AF–RE, and AF–FM–RE by interface engineering within heteroepitaxial and or polycrystalline bilayers. Exchange-biased coupling was evident for all three systems. It is negligibly weak for the AF–RE system around the temperature of the helical phase whereas it gains relatively higher values for the AF–FM–RE system around the same temperature range. We find a frustrated inter-domain magnetic interaction leading to the coexistence of spin-freezing like ordering around the temperature range of helical spin modulation at the exchange-coupled interfaces of AF–RE based specimen. Such spin-freezing phenomena cease to exist in the AF–FM–RE system, extending the SPM limit. Changing the bias direction during field cooling of the AF-coupled interface, across the intervening FM layer, we could introduce a difference in the non-collinear directions for opposite field cooling options and demonstrate its consequence on the bias field of the exchange-coupled RE layer.

## Results and discussions

### Samples

Magnetron sputtered samples on MgO(100) substrate of three different compositions are used for the study.

- Sample *S*1: [Mn$_{3}$Ir(6.0 nm)/CoFe(10.0 nm)]/TaN(2.5 nm) represents a prototypical one depicting exchange bias between AF–FM.
- Sample *S*2: [Mn$_{3}$Ir(6.0 nm)/Dy(50.0 nm)]/TaN(2.5 nm) represents a coupling between AF–RE.
- Sample *S*3: [Mn$_{3}$Ir(6.0 nm)/CoFe(10.0 nm)/Dy(50.0 nm)]/TaN(2.5 nm) represents coupling between AF–FM–RE.

### X-ray diffraction

**Figure 2:**
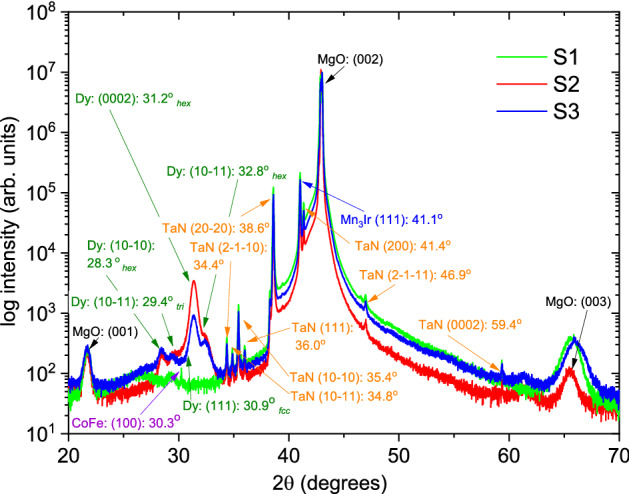
XRD for *S*1, *S*2, and *S*3. X-ray diffraction patterns of the epitaxial Mn$_{3}$Ir (*S*1, *S*2 and *S*3) and CoFe (*S*1, *S*3) indicating the main structural peaks from the layers. The Dy peaks (*S*2 and *S*3) correspond to the various polycrystalline phases. Additionally, various peaks are seen from the capping layer (TaN) and substrate (MgO).

The X-ray diffraction (XRD) patterns in Fig. 2 show the structural characterisation for *S*1, *S*2, and *S*3. All samples depict a high degree of crystallographic orientation (epitaxy) for Mn$_{3}$Ir and also for CoFe (in *S*1 and *S*3) while polycrystallinity is seen for Dy (in *S*2 and *S*3) and TaN^17^. The main peaks from the layers correspond to Mn$_{3}$Ir (002) at 46.9$^\circ$, CoFe (001) at 30.3$^\circ$, and *hex *Dy (0002) at 31.2$^\circ$.

### Transmission electron microscopy

**Figure 3:**
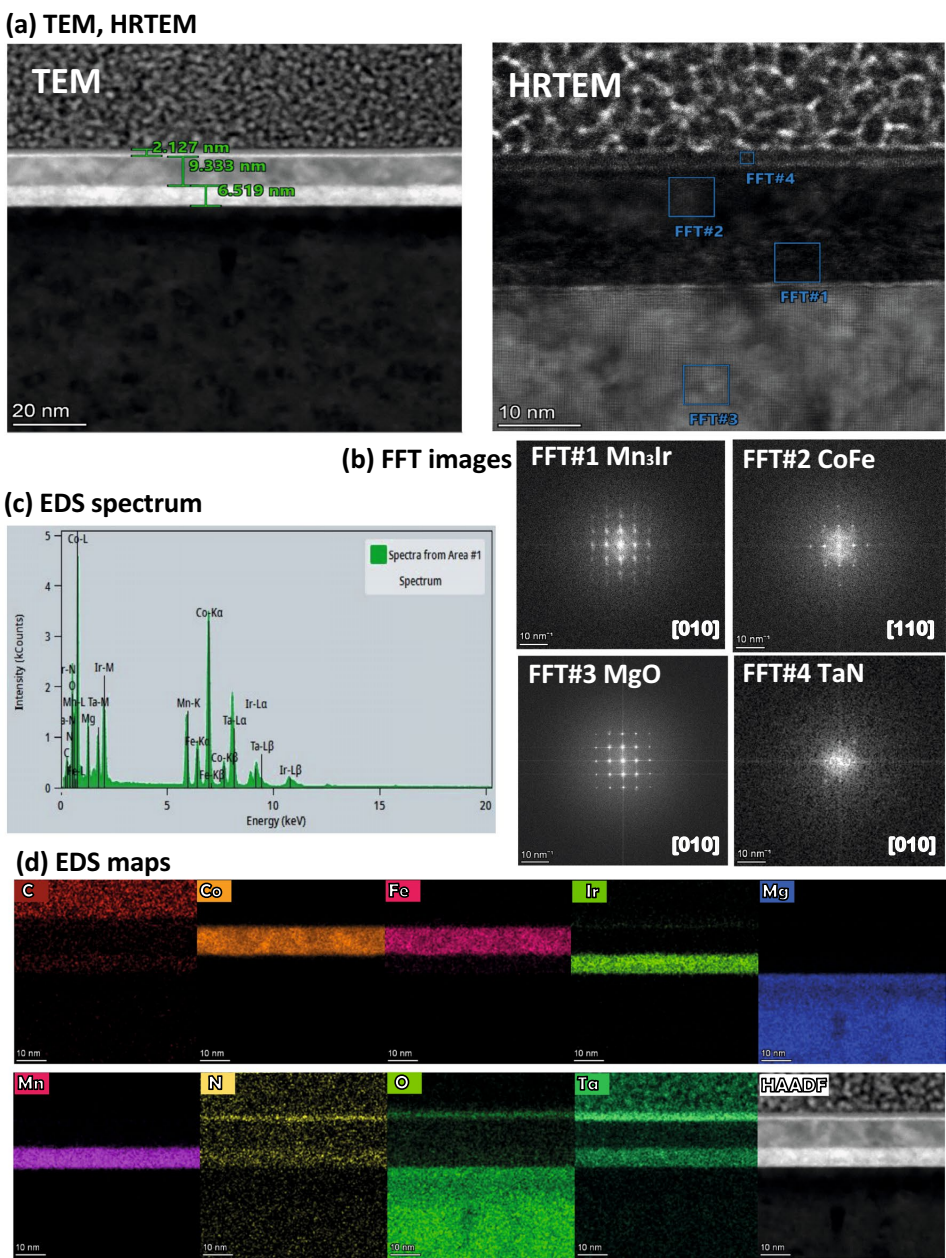
TEM and EDS for *S*1. (**a**) Cross-sectional TEM and HRTEM images of specimen *S*1, show the sequence of layers on the MgO substrate. (**b**) FFT patterns of the area marked by the squares in (**a**) containing MgO, Mn$_{3}$Ir, CoFe, and TaN. (**c**) EDS spectrum and (**d**) EDS maps of the elements C, Mg, O, Mn, Ir, Co, Fe, Ta, and N in the layer stack show sharp interfaces along with the HAADF STEM image of the interfaces. The presence of C at the top layer is from the capping layer used during sample preparation for FIB processing.

**Figure 4:**
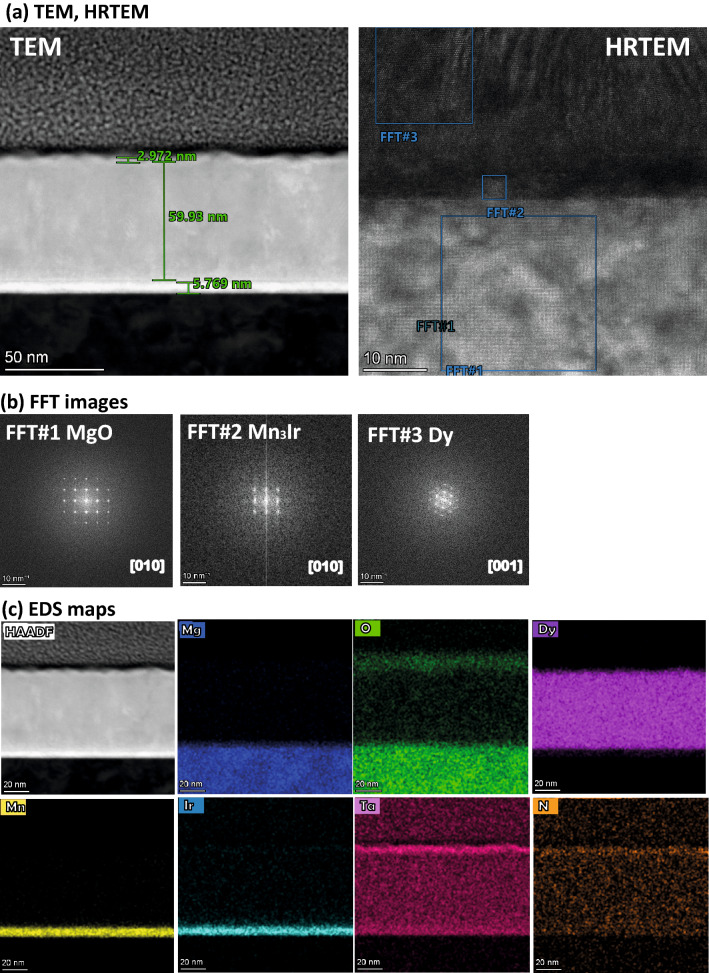
TEM and EDS for *S*2. (**a**) Cross-sectional TEM and HRTEM images of the specimen *S*2, show the sequence of layers on the MgO substrate. (**b**) FFT patterns of the area marked by the squares in (**a**) containing MgO, Mn$_{3}$Ir, and Dy. (**c**) EDS maps of the elements Mg, O, Dy, Mn, Ir, Ta, and N in the layer stack show sharp interfaces along with the HAADF STEM image of the interfaces.

**Figure 5:**
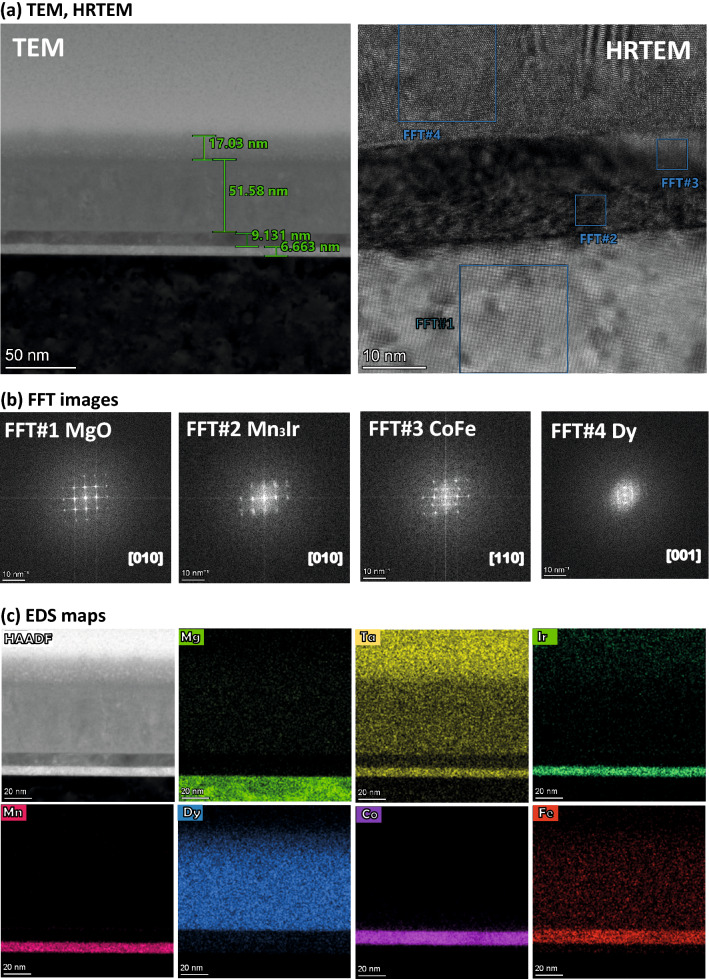
TEM and EDS for *S*3. (**a**) Cross-sectional TEM and HRTEM images of the specimen *S*3, show the sequence of layers on the MgO substrate. (**b**) FFT patterns of the area marked by the squares in (**a**) containing MgO, Mn$_{3}$Ir, CoFe, and Dy. (**c**) EDS maps of the elements Mg, Ta, Ir, Mn, Dy, Co, and Fe in the layer stack show sharp interfaces along with the HAADF STEM image of the interfaces.

Cross-sectional TEM experiments were conducted on the samples *S*1, *S*2 and *S*3 to examine the microstructure. Figures 3a, 4a, and 5a show the TEM and HRTEM lattice image along the [010] zone axis of sample *S*1, *S*2, and *S*3. The interface between MgO and Mn$_{3}$Ir is atomically abrupt and can be readily identified in all samples. A similar interface contrast between Mn$_{3}$Ir and CoFe in *S*1 and *S*3, and that between Mn$_{3}$Ir and Dy in *S*2, is also clear. The interface is sharp, suggesting very little intermixing at their interfaces.

The Fast Fourier Transformation (FFT) patterns (Figs. 3b, 4b, 5b) of the lattice images of the area containing MgO, Mn$_{3}$Ir, CoFe, and TaN for *S*1, MgO, Mn$_{3}$Ir, and Dy for *S*2, and MgO, Mn$_{3}$Ir, CoFe, and Dy for *S*3, as marked in the dashed squares in the HRTEM image of Figs. 3a, 4a, and 5a are shown alongside. The 002, 004, and 200, 400 reflections of MgO are clearly visible in all samples. On the one hand, the reflections 001, 002, and 100, 200 from Mn$_{3}$Ir are either split or superimposed, implying an in-plane strain in the films. The same reflections from CoFe (00$\bar{1}$, 00$\bar{2}$ and 1$\bar{1}$0, 2$\bar{2}$0) in *S*1 and *S*3, on the other hand, are not distorted. No such reflections can be seen for TaN, implying a disordered lattice. Ring-like reflections, signifying polycrystallinity for TaN and Dy, are observed from *S*1 and *S*3, respectively. The hexagonal reflections for Dy in *S*2 are relatively distinct as compared to that in *S*3.

We perform detailed peak analysis from the FFT spots in estimating the lattice mismatches in all samples. For *S*1, we find a little lattice mismatch ($\approx$ 5%) with MgO (001) (*a* = *b* = *c* = 4.217 Å), ensuring Mn$_{3}$Ir (001) (*a* = *b* = *c* = 4.001 Å) to remain coupled to the substrate. This is sufficient to seed cube-on-cube growth, allowing a small epitaxial strain. The interface between Mn$_{3}$Ir and CoFe lattices, on the other hand, did not see a sufficient mismatch ($\approx$ 1%), ensuring an unstrained heteroepitaxial growth. For *S*2, the MgO/Mn$_{3}$Ir interface show even smaller lattice mismatch ($\approx$ 2%) than in *S*1 and that between Mn$_{3}$Ir/Dy interface is not possible to determine due to a structural change in Dy. For *S*3, the MgO/Mn$_{3}$Ir interface again show a little lattice mismatch ($\approx$ 3%) while the Mn$_{3}$Ir/CoFe interface show significantly small lattice mismatch ($\approx$ − 0.1%). Similar to *S*2, here also, it is not possible to determine the mismatch between CoFe/Dy interface due to a structural change in Dy.

Figure 3c shows the EDS spectrum for *S*1 and the corresponding EDS maps in Fig. 3d for elemental identification across the stack while the EDs maps for *S*2 and *S*3 are shown in Figs. 4c and 5c, respectively. The layers are readily identified with sharp interfaces from the EDS maps of the MgO substrate, Mn$_{3}$Ir, CoFe, Dy, and TaN were used to reveal layer arrangement across the interfaces directly for the respective sample. Also shown are the high-angle annual dark field scanning TEM (HAADF STEM) images of the interface areas showing coherent interface features for both samples.

**Figure 6:**
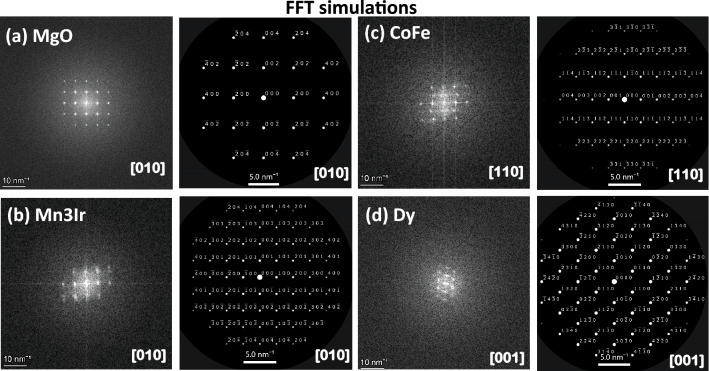
FFT patterns simulations. Simulations for the FFT patterns on a zoomed-in (5.0 nm) scale along with the FFT patterns for different zone axes: (**a**) [010] for MgO, (**b**) [010] for Mn$_{3}$Ir, (**c**) [110] for CoFe, and (**d**) [001] for Dy.

Figure 6a–d shows the zoomed-in FFT (5 nm scale) simulations for the FFT patterns in Figs. 3b, 4b, and 5b along the [010] zone axis for MgO, [010] for Mn$_{3}$Ir, [110] for CoFe, and [001] for Dy.

**Figure 7:**
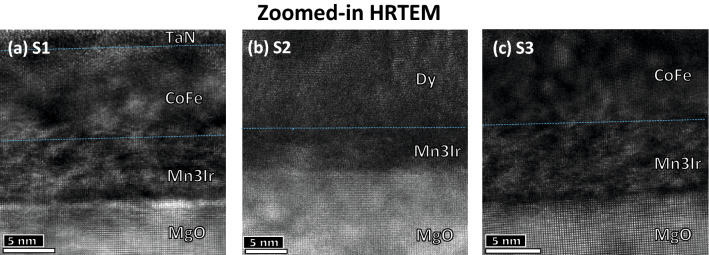
HRTEM for *S*1, *S*2, and *S*3. Cross-sectional zoomed-in HRTEM images show the sharp interfaces (rms roughness $\approxeq$ 0.2 ± 0.1 nm) of MgO/Mn$_{3}$Ir of the specimens (**a**) *S*1, (**b**) *S*2 and (**c**) *S*3.

The sharpness of the interfaces are further exemplified following the zoomed-in HRTEM images in Fig. 7a–c showing the interfaces of MgO/Mn$_{3}$Ir and Mn$_{3}$Ir/CoFe for *S*1 and *S*3, MgO/Mn$_{3}$Ir and Mn$_{3}$Ir/Dy for *S*2 with an rms roughness of $\approxeq$ 0.1–0.3 nm.

### Magnetization measurements

#### Field cooled and zero-field cooled measurements

Characterization of the magnetic properties was done using the field-dependent magnetization (*M*) measurements as a function of temperature (*T*) using field cooled (FC) and zero-field cooled (ZFC) protocols. We applied different magnetic fields $\textbf{H}_a$ = 5 Oe (0.5 mT) to 500 Oe (50 mT) during measurements after cooling down to 2 K in presence of $\textbf{H}_a$ = 5 kOe/500 mT (FC) and 50 kOe/5000 mT (FC). The same protocol was used when the samples were cooled down to 2 K in the presence of no magnetic field (ZFC).

##### Samples *S*1 and *S*2

**Figure 8:**
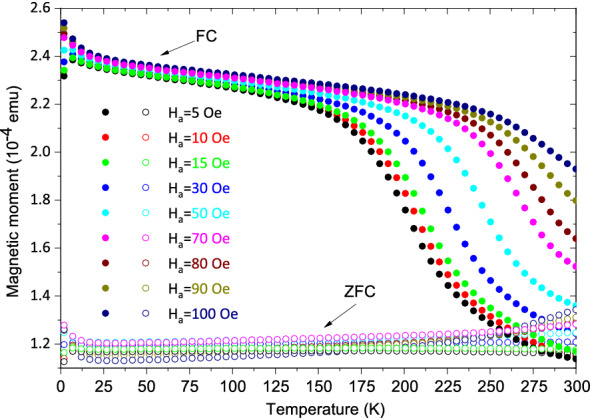
FC-ZFC measurements for *S*1. The temperature dependence of the DC magnetization. The measurements were done on heating at different fields after zero-field cooling (ZFC) and field cooling (FC) in 5 kOe (500 mT).

**Figure 9:**
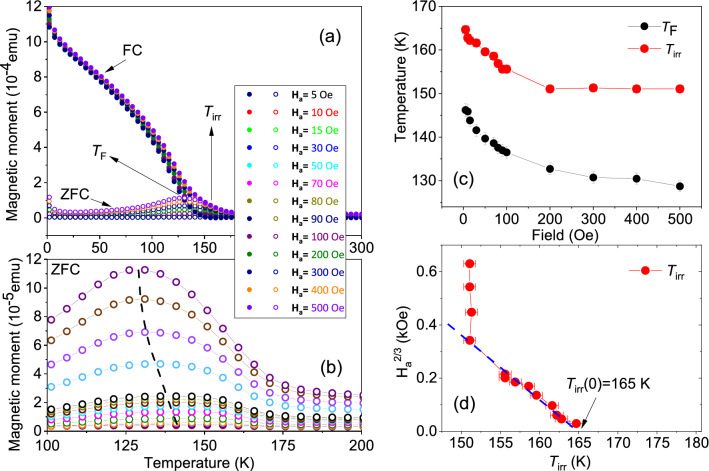
FC and ZFC measurements and $T_{{\textrm{F}}}$, $T_{{\textrm{irr}}}$ versus $\textbf{H}_a$ for *S*2. (**a**) The temperature dependence of the DC magnetization. The measurements were done on heating at different fields after zero-field cooling (ZFC) and field cooling (FC) in 50 kOe (5000 mT). (**b**) A broad maximum can be observed for the zoomed-in ZFC curves ($T_{{\textrm{F}}}$). The shift in the peak position with increasing $\textbf{H}_a$ is indicated by a dashed line. (**c**) The plot of $T_{{\textrm{F}}}$ as estimated from the ZFC curves and $T_{{\textrm{irr}}}$ versus increasing fields of measurement $\textbf{H}_a$. The lines are a guide to the eye. (**d**) The plot of $\textbf{H}_a$ $^{2/3}$ versus $T_{{\textrm{irr}}}$, which is fitted (blue dashed line) following the Almeida–Thouless (AT) equation within a limited temperature range.

The magnetization (*M*(*T*)) curves are shown in Fig. 8 for *S*1 and in Fig. 9a,b for *S*2 at various applied fields. The ZFC curves for *S*1 in Fig. 8 do not show any peak while the FC curves shift to higher temperatures with an increase in field, typical for a ferromagnetic film. One may note that the antiferromagnet (Mn$_{3}$Ir) usually having a multi-domain structure would influence FC and ZFC curves differently due to the AF–FM coupling with the ferromagnet on top (CoFe).

However, *S*2 (Fig. 9a,b) show broad ZFC maxima, which can be referred to as the blocking or freezing temperature ($T_{{\textrm{F}}}$). The appearance of a peak in the ZFC curve within a range of 70–180 K suggests that it could have an origin owed to the assembly of magnetic spin-clusters, which can pass from a blocked or frozen state (SSG) to an SPM regime with an increase in temperature. One may note that a helical structure of Dy is expected to appear within a similar temperature range (87–179 K), at least in bulk. The plot of $T_{{\textrm{F}}}$ as a function of field $\textbf{H}_a$ is shown in Fig. 9c for *S*2. We find $T_{{\textrm{F}}}$ to decrease with the field, which suggests that the frozen state is gradually suppressed by the field. With an increase in the magnetic field, as the crystal-field anisotropy starts to decrease, the thermal energy required to cross the height of energy barriers between the two easy axis orientations also decreases. A gradual convergence of the ZFC and FC curves with increasing field, indicates the attainment of a similar type of magnetic configuration near equilibrium for both samples.

The irreversibility temperature $T_{{\textrm{irr}}}$, which usually indicates the divergence temperature for FC and ZFC curves, could be seen for *S*2 to shift to lower temperatures with increasing $\textbf{H}_a$. The shift can follow the Almeida–Thouless (AT) line indicating an SSG-like behavior^18,19^. The expression for AT line is given by

1 $$\begin{aligned} \textbf{H}_a/\Delta J \propto \left( 1-\frac{T_{{\textrm{irr}}}(\textbf{H}_a)}{T_{{\textrm{irr}}}(0)}\right) ^{\frac{3}{2}}, \end{aligned}$$

where $T_{{\textrm{irr}}}$(0) is the zero-field freezing temperature and $\Delta J$ is the width of the distribution of exchange interactions. The plot of $\textbf{H}_a$ $^{2/3}$ as a function of $T_{{\textrm{irr}}}$ is shown in Fig. 9d. The curve cannot be fitted to the AT line, which separates a nonergodic (SSG) phase from an ergodic (SPM) one, except for within a limited temperature range (150–165 K) as the fitted (blue dashed) line cuts the *x* axis at $T_{{\textrm{irr}}}(0)$ $\approx$ 165 K. Thus, *S*2 shows a collective freezing behavior, which resembles spin-glass type but within a limited temperature where one expects a helical phase for Dy.

##### Sample *S*3

**Figure 10:**
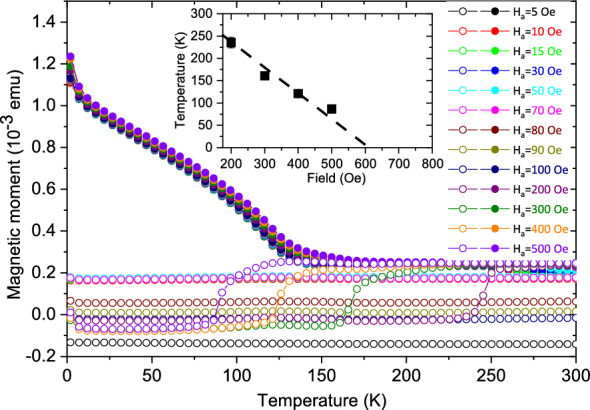
FC-ZFC measurements for *S*3. The temperature dependence of the DC magnetization. The measurements were done on heating at different fields after zero-field cooling (ZFC) and field cooling (FC) in 50 kOe (5000 mT). Inset shows the phase transition temperature of bidomains as a function of $\textbf{H}_a$.

 Figure 10 shows the *M*(*T*) curves for *S*3. The ZFC curves do not show any peak. However, at low temperatures they show negative values for $\textbf{H}_a$ $\ge$ 100 Oe. The negative values turn positive at a certain temperature, which decreases with increasing $\textbf{H}_a$ (inset of Fig. 10). Competing ferromagnetic and antiferromagnetic interactions around the energy barrier landscape lead to domains of frozen spins with opposite polarizations, which manifest themselves as coexisting bidomain states^20^.

#### Field hysteresis loops

In-plane magnetic field hysteresis loops were measured at different temperatures for *S*1 after field cooling in presence of + 5 kOe/− 5 kOe (500 mT/− 500 mT) for positive (negative) biasing and for *S*2 and *S*3 specimens at various temperatures after field cooling in presence of + 70 kOe/− 70 kOe (7000 mT/− 7000 mT) for negative (positive) biasing. The saturation field of *S*1 (*S*2, *S*3) is 2 kOe (50 kOe, 50 kOe). The remanent magnetization ($m_{{\textrm{r}}}$ = $[m_{{\textrm{r}}}^{+} - m_{{\textrm{r}}}^{-}]/2$), coercivity ($H_{{\textrm{c}}}$ = $[H_{{\textrm{c}}}^{+} - H_{{\textrm{c}}}^{-}]/2$) and exchange bias ($H_{{\textrm{eb}}}$ = $[H_{{\textrm{c}}}^{+} + H_{{\textrm{c}}}^{-}]/2$) are defined for the two branches of the hysteresis loops. The interface exchange field during the hysteresis cycle comprises of reversible ($n_{{\textrm{r}}}$) and irreversible ($n_{{\textrm{ir}}}$) components of moments for positive ($n_{{{+}}} = n_{{\textrm{r}}}+n_{{\textrm{ir}}}$) and negative field directions ($n_{{{-}}} = n_{{\textrm{r}}}-n_{{\textrm{ir}}}$) of the respective decreasing and increasing branches of the hysteresis loop. The difference in remanent magnetization $\delta m_{{\textrm{r}}}$ = $[m_{{\textrm{r}}}^{+} + m_{{\textrm{r}}}^{-}]/2$, attributed to the uncompensated spins [($n_{{{+}}}$–$n_{{{-}}}$)/2] in AF usually referred to as the vertical shift, is also defined. $H_{{\textrm{eb}}}$ is proportional to the number of uncompensated spins.

##### Samples *S*1 and *S*2

**Figure 11:**
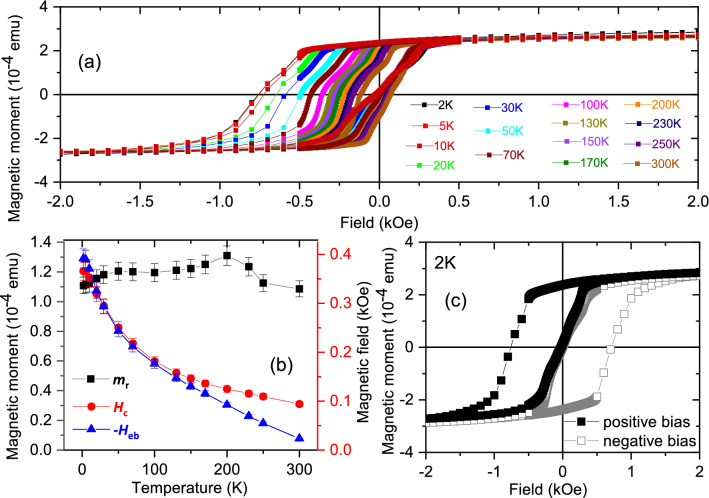
Hysteresis loops, $m_{{\textrm{r}}}$, $H_{{\textrm{c}}}$, $H_{{\textrm{eb}}}$ for *S*1. (**a**) Hysteresis loops at various temperatures showing different shifts of the loops with temperature after field cooling at a positive field. Plot of (**b**) remanent magnetization $m_{{\textrm{r}}}$, coercive field $H_{{\textrm{c}}}$, and exchange bias field $H_{{\textrm{eb}}}$ as a function of temperature. (**c**) The temperature dependence of the DC magnetization. The measurements were done on heating at different fields after zero-field cooling (ZFC) and field cooling (FC) in 5 kOe (500 mT). The CoFe saturation magnetization per unit volume estimates to $\approx$ 2.3 $\mu _B$/atom at 2 K.

**Figure 12:**
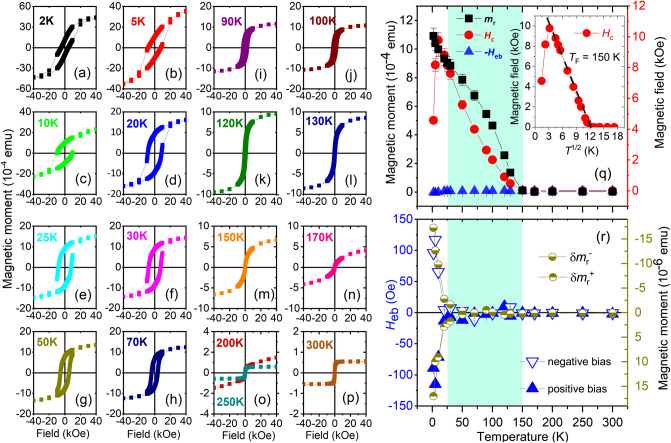
Hysteresis loops measurements and $m_{{\textrm{r}}}$ and $H_{{\textrm{c}}}$ and $H_{{\textrm{eb}}}$ for *S*2. (**a–p**) Hysteresis loops at various temperatures show the evolution of magnetization with temperature and field after field cooling at a positive field. The Dy saturation magnetization per unit volume estimates to $\approx$ 7.0 $\mu _B$/atom at 2 K. (**q**) Remanent magnetization $m_{{\textrm{r}}}$, coercive field $H_{{\textrm{c}}}$, and exchange bias field $H_{{\textrm{eb}}}$ as a function of temperature. Inset shows the $H_{{\textrm{c}}}$ versus $T^{\frac{1}{2}}$ plot and its linear fit (black dashed line) showing the maximum for the zero-field cooled (ZFC) curves $T_{{\textrm{F}}}(0)$ = 150 K. (**r**) The plot of $H_{{\textrm{eb}}}^{-/+}$ for the positive cooling field (positive bias) and negative cooling field (negative bias). The shaded region marks the temperature range of the helical phase or SPM region. Also shown are the vertical shift $\delta m_{{\textrm{r}}}^{+/-}$ values for positive and negative biasing, respectively.

 We have shown in Fig. 11a the hysteresis loops for *S*1 after field cooling in presence of + 5 kOe (500 mT) and for *S*2 in Fig. 12a–p after field cooling in presence of + 70 kOe (7000 mT), both for positive biasing. The variation of $m_{{\textrm{r}}}$ and monotonic decrease of $H_{{\textrm{c}}}$ and $H_{{\textrm{eb}}}$ with increasing *T* are plotted for *S*1 (Fig. 11b) and *S*2 (Fig. 12q). The magnetization per unit volume is $\approx$ 2.3 $\mu _B$/atom at 2 K for *S*1 while it is $\approx$ 7.0 $\mu _B$/atom at 2 K for *S*2. The bulk values of magnetization are 2.58 $\mu _B$/atom and around 2.3 $\mu _B$/atom for thin film in CoFe^21^. For Dy, they are 10.6 $\mu _B$/atom in bulk^22^ and 7.1 $\mu _B$/atom for thin film^9^.

For *S*1, neither $m_{{\textrm{r}}}$ nor $H_{{\textrm{c}}}$ (Fig. 11b) go to zero although $H_{{\textrm{eb}}}$ is seen to be gradually decreasing and tending to zero. All these are typical for an exchange-coupled ferromagnetic system. Observations of vertical magnetization shift, stemming from the antiferromagnet, are sparse as merely a fraction of the total moments in the system are participating. The temperature where $H_{{\textrm{eb}}}$ $\rightarrow$ 0, signifies the blocking temperature of the exchange-coupled system. The hysteresis loops for *S*1 showing $H_{{\textrm{eb}}}^{-/+}$ at 2 K is shown in Fig. 11c for two sets of field cooling, positive and negative, respectively. The corresponding hysteresis loops and $H_{{\textrm{eb}}}$ values are expectedly mirrored.

However, for *S*2 (Fig. 12q), both $m_{{\textrm{r}}}$ as well as $H_{{\textrm{c}}}$ go to zero, which indicate relaxation and magnetic irreversibility for *T* < $T_{{\textrm{F}}}$, the effects that are typical for supermagnetic blocked or frozen spin-clusters^23^. One may note that a monotic decrease can also be indicative of a weakness of magnetic interactions. The energy barrier against the anisotropy appropriate for SPM/SSG relaxation can be reduced by applying an external field, which effectively vanishes the magnetization at a certain field value. This field is the coercive field, which is given by

2 $$\begin{aligned} H_{{\textrm{c}}}=2\frac{K_{{\textrm{u}}}}{m_{{\textrm{s}}}}\left[ 1-\left( \frac{T}{T_{{\textrm{F}}}}\right) ^{\frac{1}{2}}\right] \end{aligned}$$

for an ensemble of non-interacting clusters of spins where $K_{{\textrm{u}}}$ is the anisotropy constant and $m_{{\textrm{s}}}$ is the saturation magnetization^24^. Thus, a linear behavior to the $H_{{\textrm{c}}}$ versus $T^{1/2}$ plot (inset of Fig. 12q) following Eq. (2) along with when $m_{{\textrm{r}}}$ tends to zero with increasing *T*, indicates SPM/SSG type of spin-clusters whereas a non-linear behavior indicates SSG type of spin-clusters. Here, a linear fit to the data (black dashed line) indicates that it is in a supermagnetic state at least below $T_{{\textrm{F}}}(0)$ = 150 K. The linearity extends up to $\approx$ 25 K. The signatures of supermagnetism is largely extended within the temperature range where one expects helical spin configuration for Dy.

The $H_{{\textrm{eb}}}^{-/+}$ as a function temperature is shown in Fig. 12r for two sets of field cooling, positive and negative, rendering the respective negative and positive exchange bias fields of equal and opposite magnitudes i.e., their values are expectedly mirrored. However, the temperature range, where a helical spin configuration of Dy is expected (marked in cyan, ranging from 25–150 K), shows meagre exchange bias fields (ranging from $H_{{\textrm{eb}}}$ = 1 ± 1 Oe to 10 ± 1 Oe), varying arbitrarily down the temperature. One may note that the helical configuration in Dy thin films, interfaced with another magnetic layer, can manifest itself within an extended or shifted temperature range than in bulk^7^. Note that the $H_{{\textrm{eb}}}$ values, within this temperature range, do not maintain their mirrored values as expected on account of their respective positive and negative biasing protocols. Interestingly, much higher $H_{{\textrm{eb}}}$ values, reaching up to − 115 ± 12 Oe/+ 117 ± 12 Oe (respectively for positive/negative biasing), can be seen at temperatures below 20 K where the Dy layer is supposed to remain in an FM state instead of a helical one. This shows that AF–RE exchange coupling is very weak in the helical phase of an RE whereas it is considerably higher in its FM phase.

The non-collinear magnetic structure was predicted to be responsible for the vertical shift $\delta m_{{\textrm{r}}}$. It was shown to be more readily observable in non-collinear in Fe/MnO$_{2}$ than in collinear AF-coupled systems^25^. Earlier, polarized neutron reflectivity revealed direct evidence of helices in the form of planar 2$\pi$-DWs within both layers of Fe and Dy^6,7^ and also within both materials of RE–RE^9^. Thus, the $\delta m_{{\textrm{r}}}$ factor, in turn, can be regarded as a footprint of non-collinearity or exchange-coupled helices within a RE-based system. Plot of $\delta m_{{\textrm{r}}}^{+/-}$ versus temperature (Fig. 12r) shows its correlation with the corresponding $H_{{\textrm{eb}}}^{-/+}$ values. In this sample there is no detectable irreversible spins ($\delta m_{{\textrm{r}}}^{+/-}$) around the temperature of the helical phase, which means the Dy interface has negligible interface magnetisation, explaining the almost disappearance of $H_{{\textrm{eb}}}^{-/+}$. Consequently, due to the weak coupling between AF–RE, the effect of non-collinear directions on $H_{{\textrm{eb}}}^{-/+}$ remains ambiguous.

##### Sample *S*3

**Figure 13:**
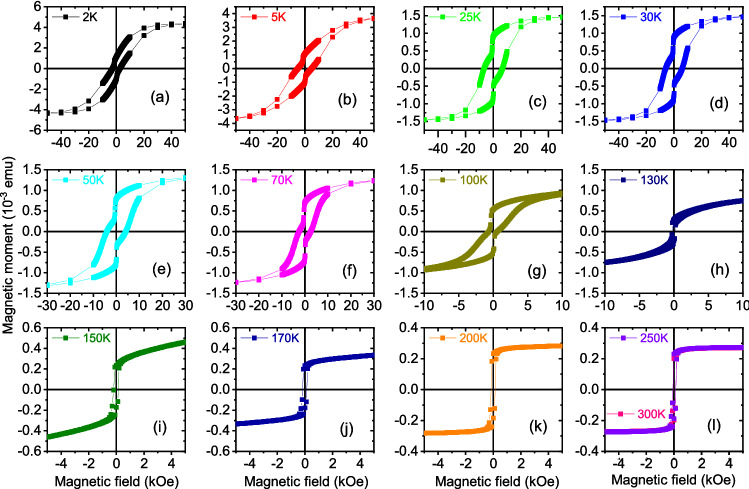
Hysteresis loops for *S*3. (**a–l**) Hysteresis loops at various temperatures show different shifts of the loops with temperature after field cooling at a positive field.

**Figure 14:**
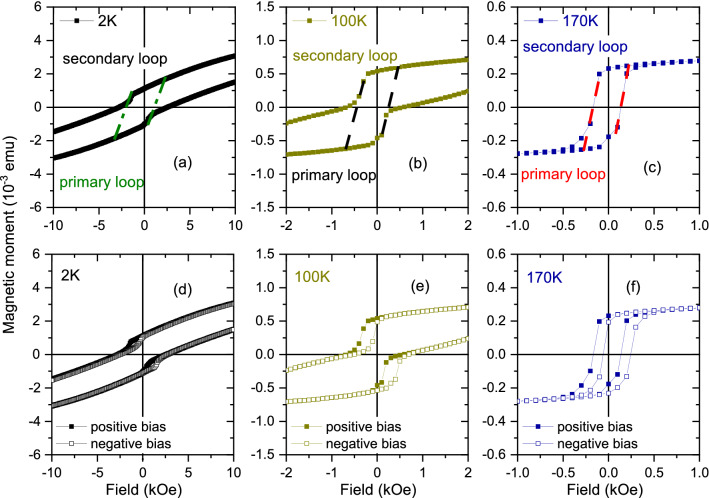
Hysteresis loop measurements for *S*3 owing to positive and negative field cooling. (**a–c**) Hysteresis loops show the coercivities of the primary and secondary loops at 2 K, 100 K, and 170 K. (**d–f**) The plot of loop shifts for positive field cooling (positive biasing) and negative field cooling (negative biasing) rendering $H_{{\textrm{eb}}}^{-/+}$ values, respectively.

The hysteresis loops of *S*3 after field cooling in presence of + 70 kOe (+ 7000 mT) for positive biasing are shown in Fig. 13a–l. Here, we see similar shifts for the bottom half (top half) along the decreasing (increasing) branch of the loops below 170 K. We categorize the hysteresis loops in terms of the superimposition of two-loop shifts: The first one is called the primary loop, centered around 0.0 Oe along the *x* axis. The second one is called the secondary loop, which has its center shifted horizontally along the *x* axis, positive and negative. Such a superposition of two loops (primary and secondary) has been reported earlier for Fe/Tb^10^ and also in Fe/Dy^7^ and was named “double hysteresis loop” (DHL). Oppositely biased subsystems with equal magnitudes of exchange bias acting on the DHLs are symmetric. Furthermore, we find a systematic variation of the $H_{{\textrm{eb}}}$ values corresponding to the shift of the primary loops as a function of temperature. Three representative double-loop characters of the hysteresis are shown in Fig. 14a–c at three different temperatures, namely 2 K, 100 K, and 170 K. We also show the effect of two sets of field cooling, positive and negative, rendering the respective negative and positive exchange bias shifts of the loops in Fig. 14d–f.

**Figure 15:**
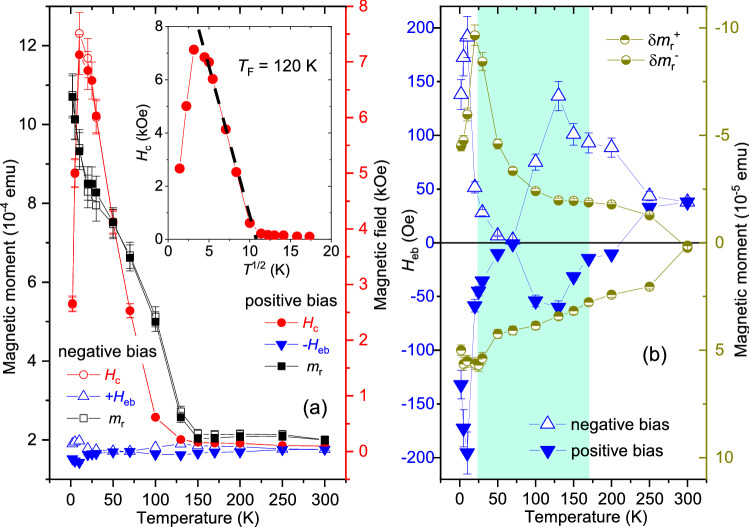
$m_{{\textrm{r}}}$, $H_{{\textrm{c}}}$, $H_{{\textrm{eb}}}$ for *S*3. (**a**) Remanent magnetization $m_{{\textrm{r}}}$, coercive field $H_{{\textrm{c}}}$, and exchange bias field $H_{{\textrm{eb}}}$ as a function of temperature. Inset shows the $H_{{\textrm{c}}}$ versus $T^{\frac{1}{2}}$ plot for positive biasing and its linear fit (black dashed line) showing the maximum for the zero field cooled (ZFC) curves $T_{{\textrm{F}}}(0)$ = 120 K. (**b**) The plot of $H_{{\textrm{eb}}}^{-/+}$s for positive cooling field and negative cooling field as a function of temperature. Also shown are the respective vertical shifts $\delta m_{{\textrm{r}}}^{+/-}$ values for positive and negative biasing.

The $m_{{\textrm{r}}}$, $H_{{\textrm{c}}}$ and $H_{{\textrm{eb}}}$ values versus temperature are plotted in Fig. 15a subjected to positive and negative biasing, respectively for *S*3. For both field cooling protocols, the $m_{{\textrm{r}}}$ values never go to zero, even though the $H_{{\textrm{c}}}$ values tend to go to zero. The non-vanishing $m_{{\textrm{r}}}$ values is due to the presence of the CoFe layer. Both these factors indicate that there is no conventional supermagnetic type of spin freezing (SSG type) or blocking (SPM type) in *S*3, which is in contrast to what was observed in *S*2 (Fig. 12q). This is also in accordance with the lack of any ZFC peak for *S*3 (Fig. 10). However, the inset of Fig. 15a show a linear behavior for the $H_{{\textrm{c}}}$ versus $T^{1/2}$ plot following eq$^{n}$ 2. A linear fit to the data (black dashed line) indicates a supermagnetic-type of state at least below $T_{{\textrm{F}}}(0)$ = 120 K extending up to $\approx$ 25 K. We believe that the supermagnetism in *S*3, which is again owed to the helical spin configuration for Dy within the temperature range, is overshadowed by the presence of the FM layer.

In Fig. 15b we show the $H_{{\textrm{eb}}}^{-/+}$ and $\delta m_{{\textrm{r}}}^{+/-}$ values as a function temperature for the two sets of field cooling, positive and negative. Higher $H_{{\textrm{eb}}}$ values, reaching up to 195 ± 20 Oe for negative/positive biasing is observed below 20 K where Dy turns FM. We find a small positive exchange bias for positive field cooling around 250 K and 300 K, which can be due to the fact that an antiferromagnetic interface monolayer reconstructs into a sufficiently rigid canted moments configuration^27^ or an AF domain wall is created within^28^, as the RE layer becomes paramagnetic.

Most interestingly, we see four important features for *S*3:

(i) Considerable exchange bias fields at temperatures (25–170 K) even where we expect the Dy helical structure to come into play.
(ii) The $H_{{\textrm{eb}}}$ values are seen to oscillate as a function of temperature till they are damped down at a certain temperature ($\approx$ 250 K).
(iii) The $H_{{\textrm{eb}}}^{-/+}$ values (positive bias/negative bias) are not mirrored, as expected, for their positive and negative counterparts.
(iv) The $\delta m_{{\textrm{r}}}^{+/-}$ values are also not mirrored and they do not follow the pattern of their corresponding $H_{{\textrm{eb}}}^{-/+}$ values.

The increased $H_{{\textrm{eb}}}^{-/+}$ values at T $\le$ 30 K is due to the ferromagnetic phase of Dy, coupled to the FM layer (CoFe). Above T $\ge$ 30 K, the exchange coupling of CoFe with Dy in its helical phase becomes evident. Plot of $\delta m_{{\textrm{r}}}^{-/+}$ versus temperature (Fig. 15b) shows its correlation with the corresponding $H_{{\textrm{eb}}}^{-/+}$ values, which is relevant here owing to its helical or non-collinear spin configuration. Earlier, Fust *et al.* have observed induced magnetic moments within the FM (Fe) layer in proximity to the RE (Dy) layer to remain either AF or FM coupled to each other depending upon the temperature and external field^7^. In the present case, an additional AF-coupling between the uncompensated moments of the AF (Mn$_{3}$Ir) with the FM (CoFe) spins exists at the AF–FM interface. All of these lead to a complex scenario where competing ferro-antiferromagnetic interactions in the system coexist. Such competition results in a compensation temperature, which is identified at 70 K where $H_{{\textrm{eb}}}^{-/+}$ $\rightarrow$ 0. Thus, instead of a direct correspondence of $\delta m_{{\textrm{r}}}^{+/-}$ with $H_{{\textrm{eb}}}^{-/+}$ as a function of temperature, we see an apparently oscillating $H_{{\textrm{eb}}}^{-/+}$, enclosed within an envelope of $\delta m_{{\textrm{r}}}^{+/-}$.

In a non-collinear structure, e.g. Dy, the pinned spins arise from the small imbalance in the number of spins in each magnetic sublattice due to the naturally occurring atomic disorder^26^. These pinned (uncompensated) spins are strongly coupled to the anisotropic bulk-like Dy helix, which explains their stability and thereby causes the $H_{{\textrm{eb}}}$ shift. These spins are also accompanied by a reversible component which explains the increase in $H_{{\textrm{c}}}$. The almost overlapping $H_{{\textrm{c}}}$ values for positive and negative biasing prove the fact that reversible components remain similar in both cases. The effect of helicity is manifested as a small statistical imbalance in the number of spins, given by $\delta m_{{\textrm{r}}}^{+/-}$, for positive and negative biasing. Thus, the $\delta m_{{\textrm{r}}}^{+/-}$ values do not overlap for opposite biasing directions. The direction and strength of the bias field depend on a vector combination of all the sublattice magnetization directions along the helix, which depends on the positions of the disordered atoms and the extent of the exchange coupling across the stack. Thus, their values do not overlap with the change in non-collinear orientations or helicity.

**Figure 16:**
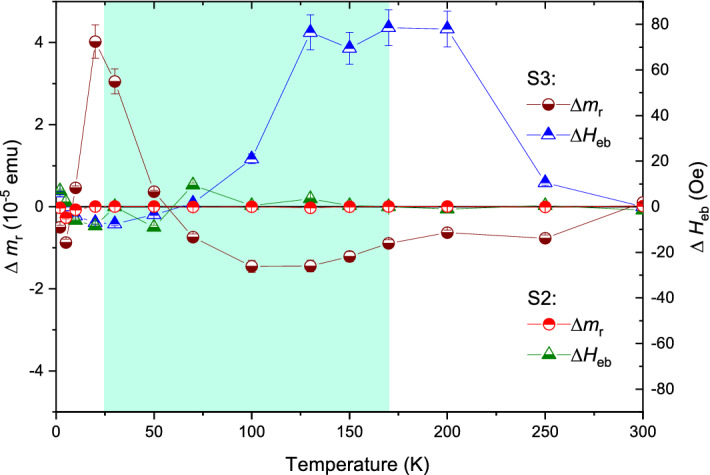
$\Delta m_{{\textrm{r}}}$ and $\Delta H_{{\textrm{eb}}}$ versus temperature. Spin imbalance $\Delta m_{{\textrm{r}}}$ and the corresponding mismatch in the bias fields $\Delta H_{{\textrm{eb}}}$ as obtained from the respective vertical shifts $\delta m_{{\textrm{r}}}^{+/-}$ and $\delta H_{{\textrm{eb}}}^{-/+}$ values and for positive and negative biasing in *S*2 and *S*3. The shaded region marks the temperature range of the helical phase of Dy.

The statistical imbalance in spin configuration $\delta m_{{\textrm{r}}}^{+/-}$ and the corresponding mismatch in the $H_{{\textrm{eb}}}^{-/+}$ values as a consequence of positive and negative biasing can be more profoundly looked into as we plot $\Delta m_{{\textrm{r}}}$ = ($\mid \delta m_{{\textrm{r}}}^{-}\mid$-$\mid \delta m_{{\textrm{r}}}^{+}\mid$) and $\Delta H_{{\textrm{eb}}}$ = ($\mid H_{{\textrm{eb}}}^{-}\mid$-$\mid H_{{\textrm{eb}}}^{+}\mid$) for *S*2 and *S*3 in Fig. 16. For *S*2, $\Delta m_{{\textrm{r}}}$ $\simeq$ 0 and $\Delta H_{{\textrm{eb}}}$ has negligible contributions. For *S*3, however, both $\Delta m_{{\textrm{r}}}$ show and $\Delta H_{{\textrm{eb}}}$ are significantly higher than *S*2 and both show a change in sign around 60 K. These higher values here, indicate a change in the spin configuration due to a change in the spin helicity imposed by the cooling protocols. One may note that we do not expect a change in the spin helicity in *S*2, since there is hardly any AF-RE coupling (nor in *S*1, as there is no RE) but is definitely expected in *S*3 (due to strong AF–FM–RE coupling) as depicted in the sketch of Fig. 1 for the two different biasing directions.

We believe the results obtained here reflect the complexity of the magnetic structures formed by the couplings of unconventional noncollinear spin structures in FM-RE with the conventional AF–FM–RE structures. Even though the non-overlapping biases indicate the fact that there exists a difference in the spin imbalance within the RE sublattice forming the helices for the two cooling conditions, more quantitative analyses based on the understanding of the exact magnetic structure would be desirable using vector magnetometric techniques like polarized neutron reflectivity. The current work remains at a rough qualitative description of the measured results with the consideration of the noncollinear magnetic structure within RE material.

## Summary and conclusion

The microscopic origin of the exchange bias effect in the prototypical AF–FM system is a small number of irreversible moments in AF sublattice (Mn$_{3}$Ir), which couples with a collinear FM (CoFe)^10^. Strong exchange bias coupling along with non-collinear spin configuration in the form of a helix has also been established in recent times for various FM–RE systems (viz., Fe-Tb, Fe-Dy)^6,7^ or RE–RE systems (viz., Dy-Tb)^9^. Owing to non-collinear magnetic sublattice in the form of a helix within an RE, exchange bias within coupled FM–RE systems is caused by a small statistical imbalance in the number of irreversible moments in RE causing a net field at the interface that pins the FM (in FM–RE) or an RE in its FM phase (in RE–RE). Notably, such an imbalance in RE cannot couple to the uncompensated moments in AF, thereby causing no exchange bias at the interface of an AF–RE system (viz., Mn$_{3}$Ir-Dy)^17^. We also find the coexistence of spin-freezing-like ordering around the temperature range of non-collinear or helical spin modulation, which is due to the frustrated inter-domain magnetic interaction at the Mn$_{3}$Ir-Dy interface.

We demonstrate here the means to overcome the absence of coupling in AF–RE by using intervening spins of FM (CoFe), which couples both, the uncompensated spins in AF (Mn$_{3}$Ir) and the non-collinear magnetic configuration in RE (Dy) within an AF–FM–RE system (Mn$_{3}$Ir-CoFe-Dy). The dependence of spin helicity or directions of non-collinearity can, in principle, be induced by equal but opposite field cooling protocols. The magnetization measurements presented here, however, are not sensitive to helicity.

The change in vertical shifts in the magnetization curves within the AF–FM–RE system is used to furthermore follow the variation of $H_{{\textrm{eb}}}$ values as a function of temperature. We demonstrate the effect of opposite field cooling protocols on $H_{{\textrm{eb}}}$ consequentially from the small statistical variation in the number of irreversible spins participating, which are distinctly different for the positive and negative fields biasing. Nevertheless in spite of the presence of the FM layer, a spin-freezing-like ordering remains coexistent for the RE layer. The enhanced understanding of controlling exchange bias by tailoring the arrangement of non-collinear magnetic spin sublattice will provide new avenues for optimizing exchange-biased systems at the nanoscale. Our findings provide not only new insights into the physical origin of exchange anisotropy at the interface of non-collinear spin structure in rare-earth and ferromagnet but also show the possibility of exploiting non-collinearity orientations as an added degree of freedom in the field of upcoming spintronic devices.

## Methods

### Sample preparation

Magnetron sputtering (DC and RF) were used to prepare the samples on MgO(100) substrate, of three different compositions. Sample *S*1: [Mn$_{3}$Ir(6.0 nm)/CoFe(10.0 nm)]/TaN(2.5 nm), sample *S*2: [Mn$_{3}$Ir(6.0 nm)/Dy(50.0 nm)]/TaN(2.5 nm) and sample *S*3: [Mn$_{3}$Ir(6.0 nm)/CoFe(10.0 nm)/Dy(50.0 nm)]/TaN(2.5 nm). Note that we have chosen a Dy thickness of 50.0 nm instead of 10.0 nm, as the magnetic moment was negligible for 10.0 nm of Dy above 35 K.

The substrates were single crystalline MgO wafers of 5$\times$5 mm$^{2}$, which were cleaned in isopropyl alcohol before use and ultrasonically cleaned in acetone and ethanol, then clamped mechanically to a holder, and subsequently heated to 250$^\circ$C under vacuum for 30 min before deposition. The targets were disks of 2 inch diameter. The thicknesses of the targets were 0.25 inch for Dy (purity of 99.9%), 0.055 inch for Co$_{80}$Fe$_{20}$ (purity of 99.95%), 0.125 inch for Mn$_{80}$Ir$_{20}$ (purity of 99.95%) and 0.125 inch for TaN (purity of 99.5%). TaN and Mn$_{3}$Ir were bonded to a copper backing plate. The targets were cleaned by pre-sputtering for 1–5 min in Ar atmosphere. The depositions were done at elevated substrate temperatures at 300$^\circ$C without post-annealing, to achieve a compromise between high quality crystal structure and a smooth surface for Mn$_{3}$Ir and CoFe. The deposition temperatures for Dy and TaN were at room temperature (RT). The deposition rates were pre-calibrated and were about 0.03 nm/s for Mn$_{3}$Ir, 0.07 nm/s for CoFe and 0.07 nm/s for Dy. The Ar pressures in the magnetron sputtering chamber were 4$\times$10$^{-3}$ mbar for Mn$_{3}$Ir, CoFe and Dy during deposition, while the base pressure was maintained at 1.6$\times$10$^{-8}$ mbar. Actual thicknesses were subsequently confirmed by measuring x-ray reflectivity, with fits to the data yielding individual layer thicknesses. The samples were grown with a high degree of crystallographic orientation (texture) for Mn$_{3}$Ir and CoFe, while polycrystallinity was obtained for Dy and TaN with an in-plane easy axis for the individual layer thicknesses chosen as a standard procedure.

### X-ray diffraction

X-ray diffraction (XRD) measurements were performed on a Rigaku SmartLab (9 kW) diffractometer at the GTIIT lab.

### Transmission electron microscopy

Specimen preparation for transmission electron microcopy (TEM) was carried out using a ThermoFisher Talos F200X at the Electron Microscopy center (GTIIT). Typical focus ion beam (FIB) Sample preparation for transmission electron microscope (TEM) was carried out using a Thermo Scientific Helios 5 DualBeam (SEM/FIB) system. Typical focused ion beam (FIB) procedures were applied to TEM sample preparation and low voltage (5 kV and 2 kV) was used for the final polishing to reduce ion-beam-related sample amorphous damage. High-resolution TEM (HRTEM) observations were conducted in a ThermoFisher Talos F200X TEM operated at 200 kV and images were recorded using Ceta 16M camera 200 kV and Velox Imaging software. Energy Dispersive X-ray Spectroscopy (EDS) experiments were conducted in a ThermoFisher Talos F200X TEM with Super-X EDS Detector. The data analysis for lattice mismatches was done using the line profiles along the spots generated by the Velox™ user interface from ThermoFisher Scientific™. The FFT simulation is generated using the SingleCrystal™ software.

### Magnetometry

Conventional in-plane magnetizations were measured at various temperatures and fields using a superconducting quantum interference device (SQUID) magnetometer from Quantum Design (MPM*S*3) at Nanomagnetism and Advanced Scattering (Nam-AST) lab (Paul’s Lab) within Guangdong Technion, Shantou.

## Data Availability

The datasets used and/or analysed during the current study available from the corresponding author on reasonable request.
